# Modularization of adhesion G protein-coupled receptor (aGPCR) structure: how alternative splicing regulates synaptogenesis

**DOI:** 10.1038/s41392-024-01829-w

**Published:** 2024-04-24

**Authors:** Tobias Langenhan

**Affiliations:** 1 Rudolf Schönheimer Institute of Biochemistry, Division of General Biochemistry, Medical Faculty, Leipzig University, Leipzig, Germany; 2 Comprehensive Cancer Center Central Germany (CCCG), Leipzig University, Leipzig, Germany; 3 Institute of Biology, Faculty of Life Sciences, Leipzig University, Leipzig, Germany

**Keywords:** Molecular neuroscience, Physiology, Cell biology

A recent report has uncovered a fascinating aspect of how neurons utilize alternative splicing of transcripts of an adhesion G protein-coupled receptor (aGPCR) gene to serve specific functions in the life of a synapse.^1^ The work provides significant insight into the ability of cells to support distinct biochemical and physiological operations through post-transcriptional processing of genetic information that is encoded in a single aGPCR locus.

Wiring a synapse is no easy task. When, where, with whom, for how long and how tight the partner neurons are fettered to one another determines the cardinal attributes of the signals they exchange, in particular during neurotransmission. Countless molecules have been associated with synaptogenesis at the two poles of a synaptic contact, the presynaptic and postsynaptic compartments. A main focus of synaptogenesis research is concerned with the question how those compartments are generated opposite each other so that the miniaturized machineries of vesicle secretion and transmitter reception align their delicate functions in space and time. Trans-synaptic protein complexes, which involve transmembrane partners inserted into the presynaptic and postsynaptic membrane, respectively, span the synaptic cleft and are a logical means to physically join and line up the two sides of a synapse. Another pivotal question asks how a synapse switches over from maturation to working mode.

Latrophilin-3/ADGRL3 (LPHN3) is a member of the large aGPCR family. These biosensors are natural chimeras that fuse structural features of GPCRs to transduce signals over the plasma membrane and feed them into intracellular metabotropic cascades with the sticky properties of adhesion proteins that fix a cell to its immediate surroundings. LPHN3 is located, among other sites in the brain, at the dendrites of CA1 hippocampal neurons, displays adhesive interplay with the presynaptic transmembrane ligands teneurin and FLRT, and its genetic corruption is associated with a host of neuropsychiatric conditions such as attention deficit hyperactivity disorder. Intriguingly, LPHN3 can engage with multiple Gα subunits, namely Gα_s_ and Gα_12/13_, which elicit fundamentally different effects in a cell. Wang et al. therefore took a closer look at how neuronal LPHN3 receptors are generated in the first place, namely how the *Lphn3* transcripts are spliced.

**Fig. 1:**
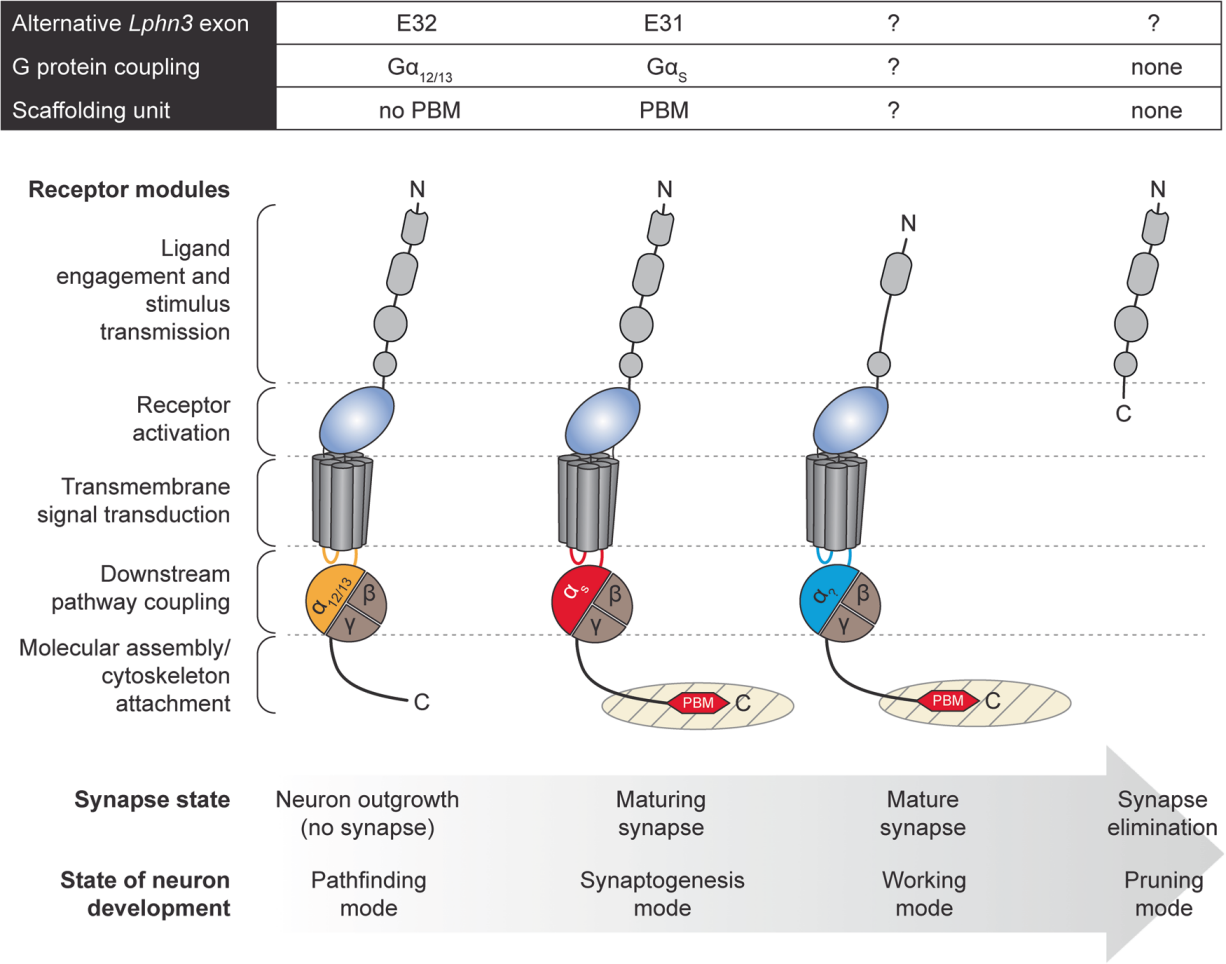
Alternative splicing of the aGPCR *Lphn3/ADGRL3* generates structurally and functionally diverse receptor variants. Summary of findings on the alternative splicing events of *Lphn3* transcripts in the mouse central nervous system (two leftmost layouts).^1^ Splicing results in the combination of structural receptor modules unique for a given *Lphn3* splice variant that determines the engagement of specific intracellular signaling and scaffolding proteins, e.g., the preferential coupling to distinct Gα subunits and PDZ domain containing proteins via PDZ binding motif (PBM). The two rightmost LPHN3 isoform layouts and their functional repertoire are hypothetical. The arrow indicates a sequence of putative developmentally regulated splicing events in accord with the ontogenetic history of an individual synapse

It was previously demonstrated that, on the whole, aGPCR family homologs undergo extensive alternative splicing generating more than a dozen splice variants per gene locus on average.^2^ However, only few studies have investigated the effects of individual splice variants and their encoded receptor isoforms. For example, alternative splicing of *GPR56/ADGRG1*, another aGPCR expressed in the brain, was linked to the evolution of cortex complexity in mammals.^3^ In the current study, alternative splice events of *Lphn3* resulted in different layouts of the receptor^1^ (Fig. 1). Selective inclusion or exclusion of exons in transcripts can therefore mold the sites in the LPHN3 receptor, where G protein coupling occurs. This pertained to the intracellular face of the heptahelical transmembrane (7TM) domain and, rather surprisingly from a structural perspective on G protein coupling, the C-terminal region of the receptor. Through pharmacological assays that tested the principal LPHN3 isoforms resulting from the splicing activity, it was shown that the most abundant E31^+^ isoform in central neurons preferentially stimulated Gα_s_ coupling, while another splice/isoform group (E32^+^) engaged Gα_12/13_.^1^ Gα_s_ activation results in the stimulation of adenylyl cyclases, which synthesize cAMP, a versatile player in a multitude of downstream pathways, while Gα_12/13_ signaling is associated with Rho GTPase activation that remodels the actin cytoskeleton. Hence, alternative splicing can determine which G protein is preferentially coupled by LPHN3 thereby potentially enabling the re-routing of the same incoming signal—relayed by engagement with its presynaptic ligand classes teneurin and FLRT—to different biochemical outputs in the postsynaptic neuron.

Wang et al. manipulated the endogenously expressed *Lphn3* splice variant pool in neuronal cultures and in mice at the expense of Gα_s_-coupling isoforms. This revealed that Gα_s_-coupling LPHN3 proteins are required for synapse connectivity, synapse density and network activity. Which synaptogenic effects are regulated by Gα_s_-stimulation through activated LPHN3 and the subsequent local production of cAMP has not been addressed in the study and will be important to follow up. In the future, the application of biosensors, e.g. to monitor G protein activity, cAMP synthesis, PKA stimulation in vivo downstream of LPHN3-proximal modifications will be necessary to capture and unravel the metabotropic effects actuated by individual LPHN3 isoforms. This may also resolve why others have found the E31^+^ LPHN3 isoform to engage with Gα_12/13_ rather than Gα_s_.^4^

However, Wang et al. uncovered another layer in the role of *Lphn3* alternative splicing for synaptogenesis. The main *Lphn3* splice variant linked to the Gα_s_-coupling receptor layout of LPHN3 also encodes a PDZ binding motif (PBM) at the C-terminus of the receptor isoform. PBMs allow to connect postsynaptic (and also presynaptic) core protein components through their PDZ domains. PBM–PDZ interactions are, thus, thought to contribute to the generation of membrane-less intracellular organelles through the process of phase separation, i.e. the formation of biomolecular condensates. It was now shown that LPHN3 isoforms cluster phase-separated postsynaptic scaffold proteins including SHANK, PSD95, GKAP and HOMER, if LPHN3 contains the PBM (Fig. 1), and that the clustering is enhanced to produce higher-order assemblies in the presence of the LPHN3 ligands teneurin 2 (TENM2) and FLRT3 in vitro.^1^ It is tempting to assume that at synaptic contacts in vivo, the formation of trans-synaptic LPHN3-TENM2/FLRT3 columns precipitate and structure postsynaptic scaffolds in a similar fashion. Remarkably, in support of this conjecture it was shown that neuronal activity correlated with a shift in splicing activity towards the generation of Gα_s_-coupling/PBM-containing *Lphn3* variants^1^ suggesting that neurons could be instructed to express synaptogenic splice variants of aGPCR, and possibly other co-factors in the process, by the synaptic signals they receive. It will also be interesting to test, which neuronal functions are supported by the Gα_12/13_-coupling LPHN3 and how their expression is controlled. Ultimately, alternative splicing of *Lphn3* transcripts may impart changes to other sites in the protein, e.g. the extracellular region, and thus display different levels of control over the biological characteristics of the receptor, e.g. its ligand binding or activation thresholds.

Pre-translational control of aGPCR functions through splicing has evolved early in evolution, and can accomplish even more dramatic changes to the layout of the gene products than the ones reported for LPHN3 in mice. For example, alternative splicing of the *Drosophila* LPHN3 homolog Cirl/CG8639 can result in the replacement of its entire 7TM domain by a single transmembrane helix, and the physiological co-expression of 7TM and 1TM Cirl isoforms titrates the sensitivity of mechanosensory neurons towards mechanical stimuli.^5^ Similarly sophisticated receptor layout changes are also predicted to occur in mammalian aGPCR transcripts,^2^ the majority of which await clarification of their biological relevance. The era of decoding the functions inscribed in the aGPCR splice repertoire has just begun.
